# Oral and vulvo-vaginal lichenoid reactions due to mitotane (Lysodren): A case report

**DOI:** 10.1097/MD.0000000000005075

**Published:** 2017-01-13

**Authors:** Arthur Schmouchkovitch, Héloïse Herry, Philippe Thuillier, Véronique Kerlan, Camille Fleuret, Guy Le Toux, Sylvie Boisramé

**Affiliations:** a Department of Dental Surgery; b Department of Endocrinology, Brest University Hospital, Brest; c Department of Dermatology, Quimper Hospital, Quimper; d Department of Oral Surgery, Brest University Hospital, Brest, France.

**Keywords:** case report, erosive lichen planus, lichenoid lesions, lichenoid reactions, mitotane, side effects

## Abstract

**Background:**

The purpose of pharmacovigilance (drug safety) is collection, detection, assessment, monitoring, and prevention of adverse effects with pharmaceutical products. It is meant to identify, characterize, prevent, or minimize actual or potential risks relating to medicinal products. To prevent these adverse effects and improve our practice, health professionals have a duty to report side effects to assess this risk and evaluate the benefit/risk requirements. Mitotane (Lysodren) is used for treating adrenocortical carcinoma. Currently, no side effects concerning oral and genital mucosa have been reported.

**Case Summary:**

This case report is about a 50 years old woman. Six months after the initiation on mitotane treatment, she developed erosive lesions located on the oral and vaginal mucosa. These drug reactions were diagnosed as erosive lichen planus by the biopsy. This lichenoid lesions were resistant to the usual treatments, mitotane being at the time not replaceable.

**Conclusion:**

This case describes an unreported adverse effect of mitotane, it is – to our knowledge – the 1st description of erosive lichenoid drug reaction due to Mitotane.

## 1 Introduction

Adrenocortical carcinoma is a cancer that develops at the expense of the outer layer of the adrenal gland. This is a rare tumor (number of new cases estimated: from 1 to 2 per million populations per year). It occurs most often in adults between 40 and 50 years old but there is also a peak incidence in children under 15 years.^[1]^ This tumor is observed most frequently in women. The treatment is complete surgical resection of the tumor, the affected nodes, and any extra adrenal lesion. Medical treatment is considered if incomplete or impossible surgery or even after apparently complete surgery (as adjuvant therapy).^[2]^

Mitotane is the generic name of 1,1-dichloro-2-(*o*-chlorophenyl)-2-(*p*-chlorophenyl)-ethane. It is an antineoplastic medication. Mitotane is an adrenal cytotoxic agent. Even if mitotane mechanisms are unknown, it seems that it slows adrenal gland function.^[3]^ It is the only adrenal-specific agent available for the treatment of malignant adrenocortical carcinoma.^[4–6]^

Oral lichen planus (OLP) is a relatively common chronic mucocutaneous inflammatory disease that affects approximately 0.1% to 2% of the population^[7]^ characterized by a disorder of keratinization, and whose aspects are polymorphic. It can reach the skin, skin appendage, and squamous mucosa (oral, genital, anal, and conjunctival mucosa). It can affect all areas of the oral mucosa but the posterior inferior cheek region represents the elective site. Localized forms are more common than diffuse forms and are frequently bilateral and roughly symmetrical. Pathogenesis is still unclear. Intense stress or anxio-depressive state, diabetes, and hypertension are often reported as trigger factors. OLP could have various etiologies: psychosomatic form, secondary form associated with a general pathology, and secondary form from graft versus host disease.^[3,7]^ Moreover, “induced” lichen planus is described and is linked to medicinal or toxic forms. Drug etiology should be sought and eliminated in front of histological lichen planus diagnosis. But interruption of treatment does not necessary conduct to improvement. This “lichenoid lesions” or “lichenoid reactions” are clinically and histologically identical to OLP. They can be due to systemic drug or dental restorative materials (gold, mercury, chromium, and copper sulfate).^[8,9]^ Drugs most frequently implicated are nonsteroidal antiinflammatory and angiotensin-converting enzyme inhibitors. Other drugs known to cause this lichenoid reactions include antimalarials, antidepressants, drugs used in treatment of rheumatoid arthritis (gold salts, D-penicillinamine), antihypertensives, diuretics, beta-blockers, hypoglycemic drugs, some antimicrobials, and many others and the list lengthens frequently.^[10,11]^

To our knowledge, we report the first case of oral and vulvo-vaginal erosive lichenoid eruptions due to mitotane.^[12,13]^

## 2 Case report

We report the case of a 50 years old woman followed in the Department of Endocrinology at Brest University Hospital (France) for malignant adrenocortical carcinoma.

In October 2013, the patient came to the emergency department for a superficial phlebitis of the left calf associated with dyspnea. Additional tests revealed a large mass of 6.6 cm long axis at the expense of the left adrenal gland. Faced with this suspicion of adrenocortical carcinoma, the patient underwent resective surgery, planned quickly. Pathological analysis confirmed the diagnosis of malignant adrenocortical carcinoma.

As part of the treatment, the patient received mitotane (Lysodren) as adjuvant treatment. Six months after starting the treatment, she reported severe discomfort at vaginal and oral levels (causing difficulty feeding). An alteration of the mucous membranes was observed. Mitotane treatment also resulted in a central hypothyroidism after 1 year of treatment. This side effect was treated by levothyroxine sodium (Levothyrox). The mensual plasma dosage of mitotane confirmed the stable level of the drug between 14 and 20 nanograms per milliliter, as recommended.

The initial diagnosis, established in the Endocrinology Department from the clinical data, was an oral candida fungus treated by flucytosine (antifungal for systemic use indicated in severe fungal infections) and fluconazole (antifungal indicated especially in the treatment of candidiasis). Due to lack of efficiency, patient was addressed in the Dermatology Department where a possible lupus was discussed. Immunological tests and vaginal and oral biopsy were performed. A presumptive diagnosis of lichen planus led to corticosteroids mouthwash prescription. Presentation of this case was made in the multidisciplinary lupus consult. This staff concluded that clinical and immunological examinations were not in favor of an induced lupus. The treatment then undertook was mouthwashes of lidocaine (local anesthetic) and prescription of Colchimax (consisting of methyl tiemonium, opium, and colchicine: antigout and antiinflammatory especially indicated to treat Behçet disease). One month later, treatments were suspended because of healing courses of lesions. Investigations have also been undertaken to rule out a deficiency origin. Vitamin supplements were prescribed.

Finally, biopsy performed at vaginal level revealed an active erosive lichen (Fig. 6). Oral biopsy is not specific but by analogy the diagnosis was bipolar erosive lichen planus. The lichen was first treated with corticosteroids (prednisone) that have proven ineffective and badly tolerated by the patient. Then treatment with ciclosporine (Neoral: immunosuppressive therapy) was conducted to the same result. So, prescription of Acitretin (Soriatane: keratolytic agent indicated for the treatment of severe forms of lichen planus on failure of the usual therapeutic) without improvement.

**Figure 1:**
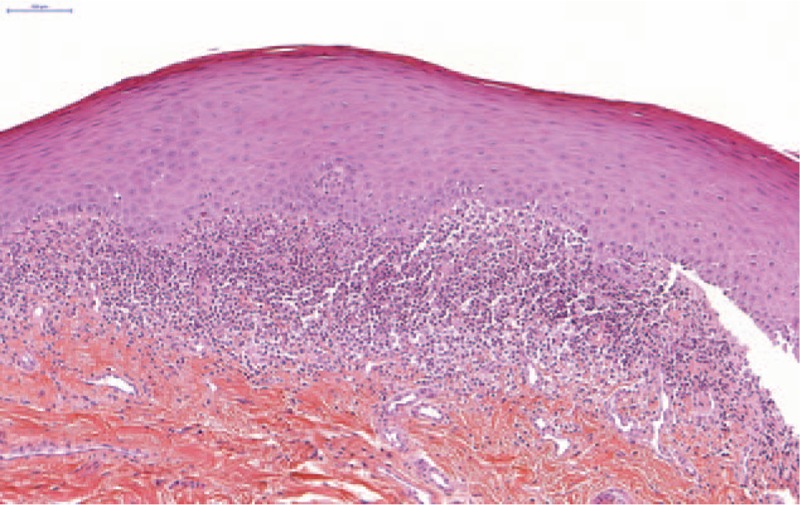
Histological section from biopsy, Hematoxylin Eosin Saffron (HES) staining, magnification 200.

**Figure 2:**
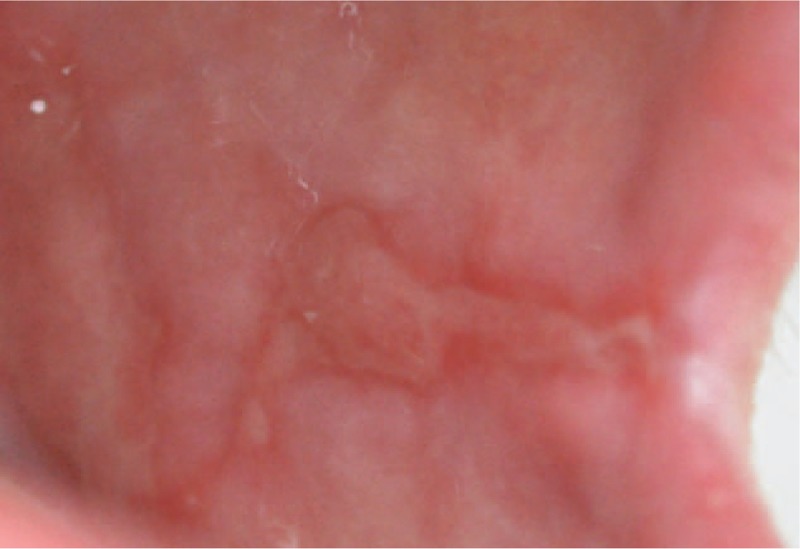
Erosive lichenoid eruption on right cheek.

The patient was sent to the Pathology and Oral Surgery Department of where were objectified:

Erosive lichenoid lesions on right and left cheeks (Figs. 1 and 2).

**Figure 3:**
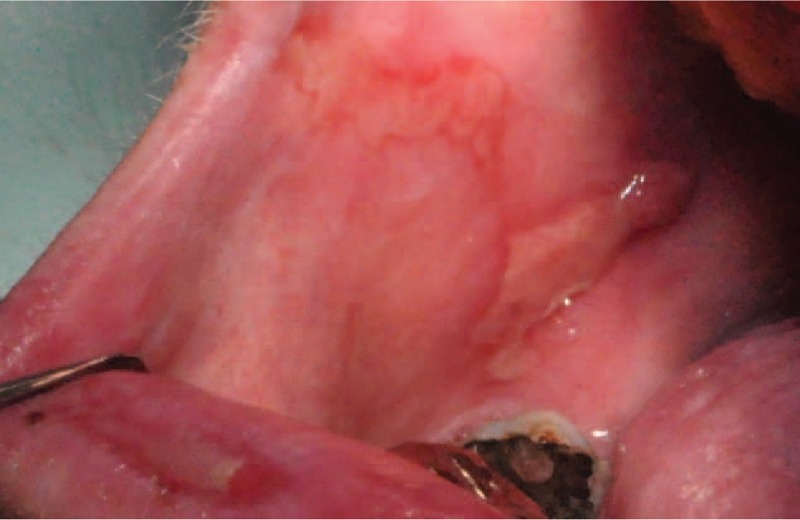
Erosive lichenoid eruption on left cheek.

Erosive lichenoid lesion of the right side edge of the tongue (Fig. 3).

**Figure 4:**
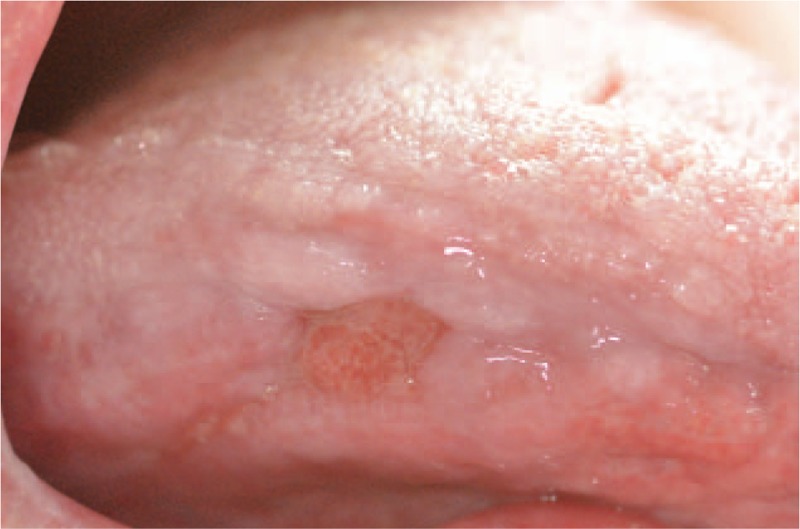
Erosive lichenoid eruption on right side of the tongue.

Complicated with pyogenic granuloma on left cheek (Fig. 4).

**Figure 5:**
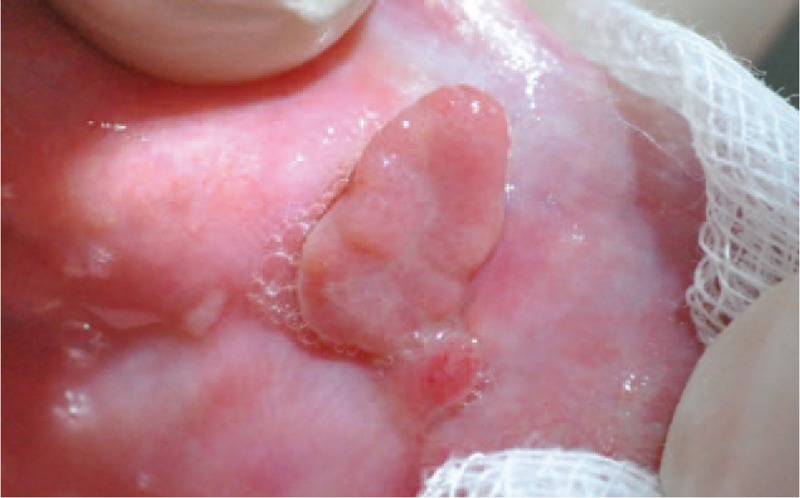
Pyogenic granuloma on left cheek.

Erosive lichenoid eruptions associated with endovaginal synechia (Fig. 5).

**Figure 6:**
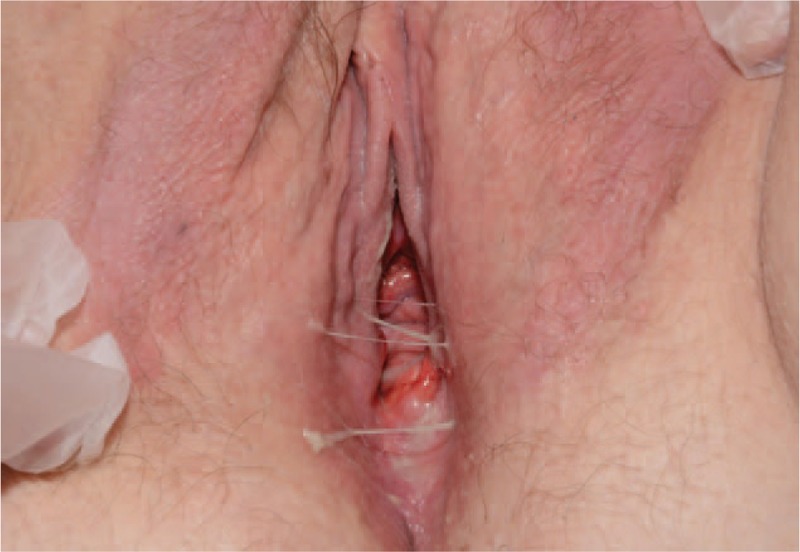
Erosive lichenoid eruption with endovaginal synechia.

Now, the patient is treated – for its erosive lichen planus – by hydroxychloroquine sulfate (Plaquenil: indicated for the treatment of lupus erythematosus and steroid-resistant or refractory lichen planus) associated with corticosteroids mouthwash for OLP and clobetasol propionate (Dermoval) with cortisone for vulvo-vaginal eruptions. At its last consultation, the patient reported significant improvement in both oral and vaginal symptoms.

Currently, discussion is ongoing to evaluate the benefit/risk balance for the maintenance or not treatment of mitotane, medication being not substitutable at the time.

## 3 Discussion

Lichenoid lesions correspond to lesions that are clinically and histologically similar to OLP. In contrast of the idiopathic nature of OLP, oral lichenoid lesions are often associated with a known identifiable cause. Lichenoid reactions have been reported after the 2nd world war with the use of antimalarial drugs (mainly quinacrine) then cited by Almeyda and Levantine.^[12]^ Since, many substances were associated with these lesions. Serrano-Sànchez et al (2010)^[13]^ proposed a review of the literature of different drugs that may cause these lichenoid reactions. We completed this review and proposed a table with different drugs incriminated in oral lichenoid lesions (Table 1). It may be noted that 20% to 25% of women with oral lichen planus have vulvovaginal involvement, generally in erosive or desquamative form.

**Table 1:**
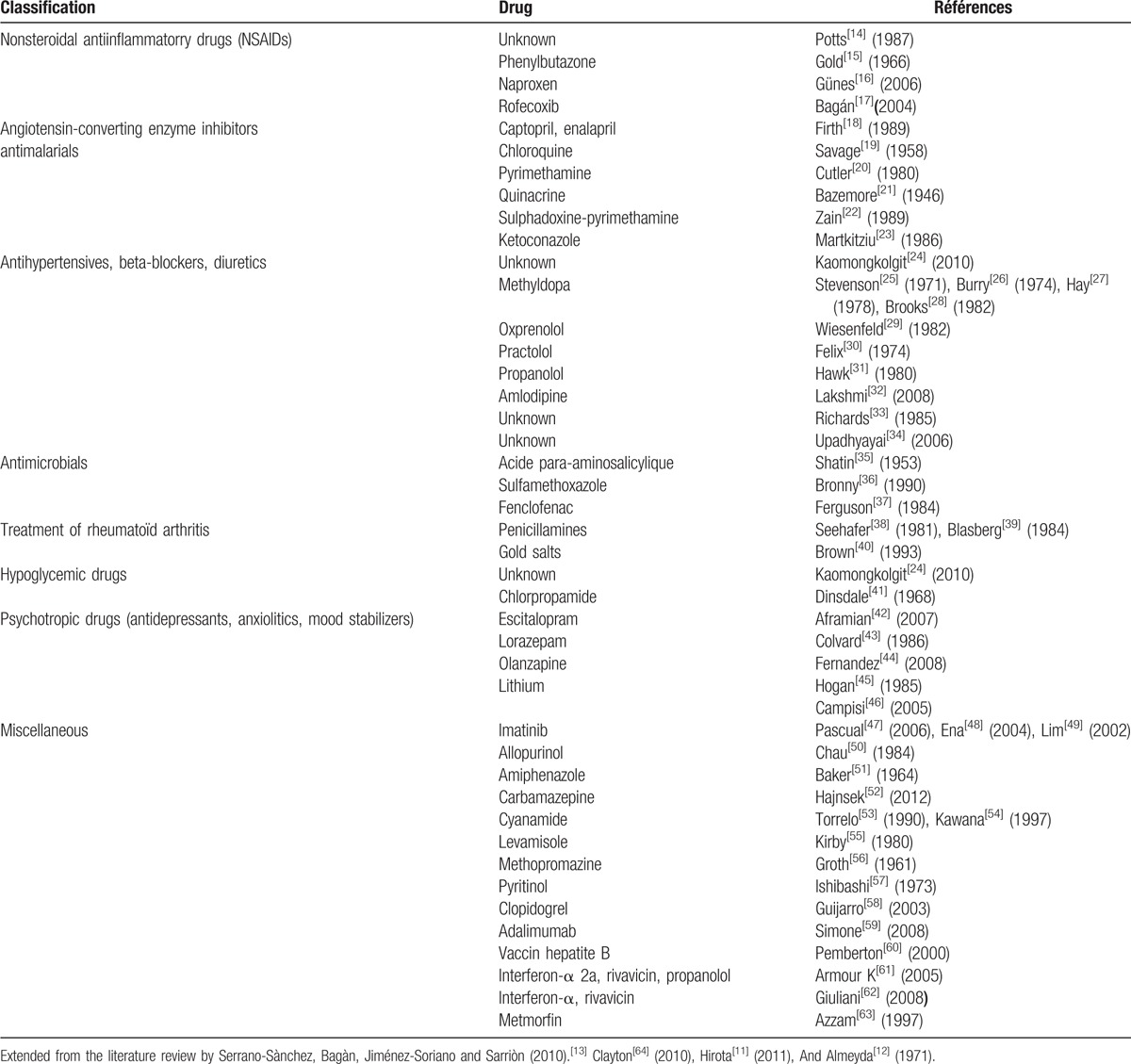
Lichenoid drug reactions in literature.

The etiology and pathogenesis of lichen planus is still unknown. Latest hypothesis refers to a probable genetic background and an autoimmune action with a lymphotoxicity reaction against basal membrane. Some factors are associated with the disease: autoimmune disorders (such as chronic active hepatitis, ulcerative colitis), diabetes and hypertension, stress and anxiety, trauma, food allergies, habits like cigarettes or betel nut chewing, malignant neoplasms, bowel disease and more commonly infectious agents (such as *Helicobacter pilori*, Epstein–Barr virus, human herpes virus 6, human immunodeficiency virus, and hepatitis C), and drugs.^[65]^ For didactic purposes, an updated table of lichenoid drug reactions is proposed (Table 1).

To our knowledge, this is the first reported case on oral and vaginal lichenoid reactions due to mitotane. Mitotane is known to cause various skin rashes but no cases of oral or genital erosive eruption had been described. The biochemical mechanism of mitotane is unknown. It has not been studied in a clinical development program. The available clinical data are largely derived from published data in patients with inoperable or metastatic adrenal carcinoma. In terms of overall survival, 4 studies concluded that mitotane does not increase the survival rate, while 5 reported an increase survival rate.^[3,66–68]^ Available data suggest that mitotane modifies the peripheral metabolism of steroids by directly suppressing secretion of the adrenal cortex. The administration of mitotane alters the extra-adrenal metabolism in humans, leading to a decline of measurable 17-hydroxy corticosteroids, even without decrease in plasma corticosteroids. Mitotane apparently causes increased formation of 6-beta-hydroxy cholesterol.^[69]^ Recent studies showed that mitotane directly interacts with lipid membranes by intercalation of mitotane into phospholipidic bilayers impacting on membrane.^[70]^ The most common side effects are gastrointestinal disorders (inflammation of the lining, vomiting, diarrhea, nausea, and epigastric discomfort), neurological side effects (ataxia, paraesthesia, dizziness, drowsiness, mental impairment, polyneuropathy, motor disorder, headache, and confusion), hematological and lymphatic disorders (leukopenia, prolonged bleeding time), musculoskeletal disorders (myasthenia gravis), metabolic and nutritional disorders (anorexia, hypercholesterolemia, and central hypothyroidism),^[71]^ general disorders (asthenia), and as conditions of the skin and subcutaneous tissue (skin rash). Lesions linked to lichen planus have been described in cutaneous^[72]^ level but not on the oral and genital mucosa.

Typically the treatment of these lesions involves stopping the substance. A therapeutic window has been considered in consultation with the Department of Endocrinology in order to highlight the correlation between mitotane and erosive lesions. However, mitotane still irreplaceable at this time for the management of malignant adrenocortical carcinoma. This constitutes a limitation of this ascertainment.

After various consultations (endocrinology, dermatology, and oral pathology), probable diagnosis was eliminated in favor of bipolar erosive lichen planus in connection with mitotane introduction 6 months before. Other explanations were eluded. In fact, paraneoplastic lesion was eliminated by anatomopathological exam. Contact dermatitis has been removed by the dermatologist. In addition, idiopathic origin would not have been refractory to treatment.

This patient is under antihypertensive therapy since several years. But mitotane was the only new drug introduced prior to lichenoid reactions development. Moreover a case of cutaneous lichen linked to mitotane has previously been reported in the literature.

This presents real challenges for the care of the patient regarding her life comfort, as far these lichenoid reactions were resistant to all therapeutics implemented. However, an improvement was observed with treatment by hydroxychloroquine sulfate (Plaquenil) efficacy already observed in 1993 by Eisen in an open trial conducted with ten patients who had biopsy-proven OLP.^[73]^

## 4 Informed consent

The patient gave her consent to publish this case report.

## 5 Conclusion

Mitotane should now be registered in the list of substances causing lichenoid lesions on oral and vaginal mucosa. This first case report should be known by medical population (endocrinologists, dermatologists, oral, and dental surgeons) to improve the therapeutic care.
